# The Analysis of Mechanical Properties and Geometric Accuracy in Specimens Printed in Material Jetting Technology

**DOI:** 10.3390/ma16083014

**Published:** 2023-04-11

**Authors:** Natalia Majca-Nowak, Paweł Pyrzanowski

**Affiliations:** 1 Łukasiewicz Research Network–Institute of Aviation, al. Krakowska 110/114, 02-256 Warsaw, Poland; 2 Institute of Aeronautics and Applied Mechanics, Warsaw University of Technology, Nowowiejska Str. 24, 00-665 Warsaw, Poland; pawel.pyrzanowski@pw.edu.pl

**Keywords:** additive manufacturing, Material Jetting, PolyJet, MultiJet, Rigur, acrylonitrile butadiene styrene (ABS), Durus, Vero, VisiJet, tensile behavior, layer height, layer orientation

## Abstract

The purpose of this research was to analyze polymer materials based on mechanical properties and geometrical parameters, such as the smallest material deviations and the best printing texture after three-dimensional (3D) printing in two methods of Material Jetting technology: PolyJet and MultiJet. This study covers checks for Vero Plus, Rigur, Durus, ABS, and VisiJet M2R-WT materials. Thirty flat specimens were printed both for 0 and 90 raster orientations. Specimen scans were superimposed on the 3D model from CAD software. Each of them was tested, paying attention to the accuracy and the layer thickness effect of printed components. Then, all specimens were subjected to tensile tests. The obtained data—Young’s modulus and Poisson’s ratio—were compared using statistical methods, focusing on the two most important parameters: the isotropy of the printed material in two directions and the characteristics close to linear. It was found that unitary surface deviation with general dimensional accuracy equal to ±0.1 mm was the common feature of printed models. Some small areas had lower accuracy depending on the material and printer device. Rigur material obtained the highest mechanical properties. Dimensional accuracy in Material Jetting technology as a function of layer parameters such as layer thickness and raster orientation was checked. The materials were checked in terms of relative isotropy and linearity. Additionally, similarities and differences between PolyJet and MultiJet methods were covered.

## 1. Introduction

The durability and mechanical properties of polymer have been improved by the latest research and by using advanced technology [1,2,3,4,5]. The reason for this is that some end-use applications are suitable through using three-dimensional (3D) printing technology [6,7,8]. The significant advantage associated with Additive Manufacturing (AM) technique is that the cost and production time do not increase with the degree of complexity [9]. The main disadvantage of AM is the anisotropic mechanical behavior of components which is mainly caused by the type or parameters process, deposition strategy, and pore distribution [10,11].

Based on the literature, the type of technology, component material, support material, the thickness of the layer, raster orientation, positioning of the specimen on the printing table, printing temperature, curing time, working speed, infill pattern and density, and the humidity of the working environment have the impact on the material mechanical properties [5,12,13,14,15,16,17,18]. The influence of raster orientation on mechanical properties is a frequent issue in the literature [19]. The research [20,21,22,23] proves that it has no significant influence on Material Jetting technology (MJT).

Material Jetting technology includes two main methods: MultiJet (MJ) and PolyJet (PJ) [24,25,26]. Both technologies use UV light to cure photopolymer extruded from the piezoelectric head in the shape of fine drops [27]. Support material is extruded from another head, and after finishing the 3D printing process, it is removed [28]. The MJT includes a wide range of polymers [29,30,31]. One of the main differences between MultiJet and PolyJet is the method of removal of the support structure. In MultiJet, the support material is wax, which has a lower melting temperature than the part material, and thus, it is melted out in an oven. However, in PJ, there are diverse support materials that are removed by using water under high pressure, a mechanical method, or an alkaline solution.

The MJ method is used to create rubber or hard plastic parts for diverse applications, including the possibility of producing wax patterns for lost-wax castings of midsize and large foundry applications [32]. The MultiJet method is checked considering the printing feasibility of airfoil wind tunnel shape [33]. According to Olasek and Wiklak [33], it is discovered that only the MultiJet printing method enabled 0.4 mm cooling holes, which is evidence of the high accuracy of this method. MJ uses an acrylic photopolymer, including grades such as ABS, Polycarbonate, and Polypropylene, known as the VisiJet materials family. The nominal accuracy by 3D Systems is between 0.025 mm and 0.05 mm per 25 mm of part dimension.

PJ is a proprietary method by Stratasys [34] and is precisely described in the patent EP 1274551 B1 [35]. Stratasys declares that the PJ method features 0.016 mm layer thickness with accuracy as high as 0.1 mm for smooth surfaces, thin walls, and complex geometries [36]. According to Yoo et al. [37], models fabricated by the PJ technique showed significantly higher accuracy, including the smoothest surface in comparison with digital light processing (DLP) and stereo-lithography (SLA) models. Results obtained during an investigation by Kluska et al. [38] for 0.028 mm layer thickness revealed that simple objects, like dumbbell specimens, could be printed with ±0.05 mm accuracy, whereas complicated shapes can achieve results ±0.15 mm. The PJ method uses “FullCure” materials, which include different properties, opacity, and flexible characteristics [39]. The “FullCure” family consists of five different material groups such as FullCure705, FullCure720, Vero, Durus, and Tango materials family [40]. The nominal accuracy is up 0.2 mm and between 0.2 mm and 0.9 mm for features below 50 mm. One of the most common materials used in the PJ method is Vero White Plus (RGD835) which is opaque, white in color, rigid, durable, and has excellent detail visualization. For specimens printed of Vero White Plus material, macrostructure observations revealed smooth and straight crack edges. Fracture is characterized by a cleavage appearance, which is a common feature of brittle materials [41]. Mechanical properties of the Vero White Plus indicate an appropriate response for biocompatibility in modeling infant skulls based on research by Khalid et al. [42]. The Stratasys data sheet for Rigur (RGD450) material (Durus materials family) informs that it is an advanced, simulated polypropylene with high toughness, excellent dimensional stability, and surface quality. Durus White (RGD430) material’s main purpose is to prototype semi-rigid polypropylene products which can withstand contact forces due to their high toughness. Digital ABS (RGD5160–DM) material is fabricated from RGD515 and RGD535. It is designed to simulate ABS engineering plastics by combining high-temperature resistance with high toughness. Digital ABS is suitable for any simulated parts that require high-impact resistance and shock absorption.

The aim of this paper is to study these two methods (MJ and PJ) and further investigate their potential in the additive manufacturing of complex 3D models, such as airplane structures that require high dimensional stability, mechanical strength, and isotropic behavior. This is a general problem of polymer 3D printing, where manufacturers focus on the exact representation of the shape of the model rather than on maintaining repeatable and equal mechanical properties of the model. In addition, the anisotropy of 3D prints introduces the difference between the quality modules in the compliant and perpendicular orientation to the direction. Research is focused on finding a material among Vero White Plus, Rigur, Durus White, ABS Ivory GEN 2, and VisiJet M2R-WT which will be suitable for printing and testing complex geometries, where isotropy of the material is an important parameter throughout a complex 3D model.

## 2. Materials and Methods

**Figure 1:**
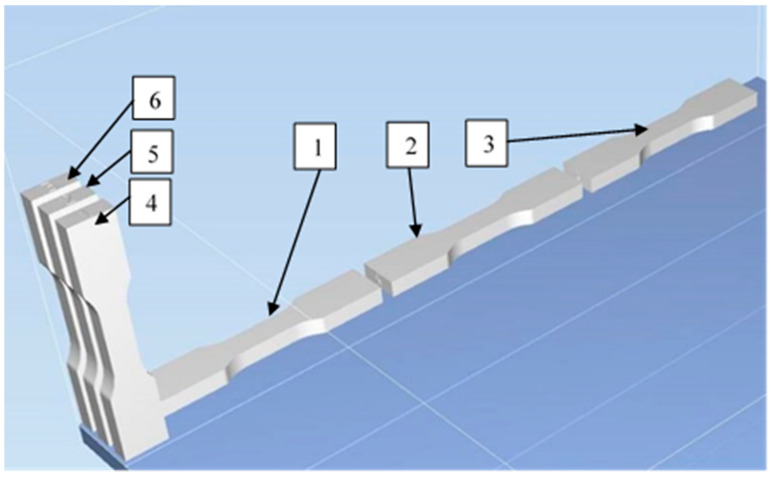
Specimen arrangements during the printing process; three (1–3) printed horizontally (X) and three (4–6) printed vertically (Z).

For analysis covered in this paper, CAD models in the shape of flat specimens designed according to the ISO527 standard were prepared. The specimens were adapted to the capabilities of available testing machines and thickened to 10 mm. All specimens had the same shapes, and their measurements were length: 140 mm, thickness: 10 mm, and width: 15/25 mm (Figure 1). In the first step, 30 specimens in five printing processes were printed in MJ and PJ methods to compare raster orientation, varied materials, and methods. The PJ method used “FullCure” materials which include different properties, opacity, and flexible characteristics; therefore, four different materials were considered. The MJ method used the VisiJet materials family, which includes similar properties; therefore, one material was considered. In addition, it is assumed that six specimens for each process constitute a representative probe to be able to perform statistical analysis. A minimum of five specimens should be tested for statistical analysis. Due to the cost of printing specimens and performing tests, it is considered that six specimens were sufficient to perform the statistical analysis presented in this article. All specimens were numbered from 1 to 6. Half of the specimens were printed horizontally (on the X axis), and others were printed vertically (on the Z axis). An example of specimens’ arrangements during the printing process is shown in Figure 1.

For the first step, in the MJ method, six specimens were printed on ProJet MJP 2500 3D printer from VisiJet M2R-WT material with a nominal layer thickness of 0.032 mm. In the PJ method, 24 specimens were printed on the Objet Connex3 3D printer from four materials: Vero White Plus, Rigur, Durus White with a nominal layer thickness of 0.016 mm, and ABS Ivory GEN 2 with a nominal layer thickness of 0.03 mm. Moreover, the SUP 707 water-soluble support material was used. After the printing process, the support material is only rinsed from the specimens, so no additional work is performed. In order to determine the cross-sectional area of the samples, thickness and width measurements are made.

In the second step, 2 out of 6 specimens from each set were selected; one specimen was printed on the X axis (ID specimen: 2) and one printed on the Z axis (ID specimen: 5), and these specimens were scanned. The first parameter is the subject of further comparisons, checking the geometrical correlation between specimens printed in two directions: vertically (along the Z axis) and horizontally (along the X axis). Based on that, a 3D Steinbichler Comet L3D scanner with a nominal measurement accuracy of the machine equal to 0.01 mm was used. Then, the specimens’ scans were superimposed on the 3D model from CAD software (Simenes NX 12) to verify a dimensional deviation. The specimens’ scans show average values of the deviation, and they do not distinguish the results with respect to the axis. The results consider both the allowances and the losses of material.

In the next step, all specimens were subjected to a tensile strength test until they were broken and material properties were determined.

**Figure 2:**
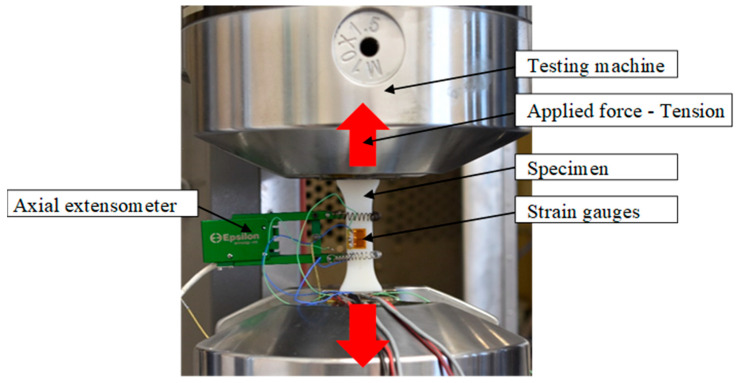
The specimen was mounted in the machine during the tensile test.

The tests were conducted in accordance with ISO 527-1: 12 on the MTS Landmark 370.1 testing machine with strain gauges to measure both axial and transverse deformations. Axial extensometer EKS025-1 is used to calibrate the machine and strain gauges. A specimen mounted in the machine during the test with an extensometer and strain gauges is shown in Figure 2.

On the basis of the tests performed, Young’s modulus and Poisson’s ratio, as well as the maximum load and tensile strength, were determined for each specimen. Additionally, their average values, standard deviation, and coefficients of variation were calculated for each process. The Young’s modulus from the slope of the strain–stress curve and the data from a strain gauge ranging from 1000–3000 strains were used for the calculations. The stress was calculated in relation to the force and the cross-section of the sample. The width and thickness were measured in the center of the sample. For the calculations, the result is the average of three measurements.

The second parameter, which is the subject of further comparisons, checks the mechanical correlation between specimens printed in vertical and horizontal directions. Based on that, statistical analyses were carried out to verify hypotheses of isotropic material properties. In order to compare Young’s modulus with regard to raster orientation, average values of Young’s modulus were determined for horizontally and vertically printed specimens, respectively. It was checked whether the analysis of outliers and the data distribution were close to the normal distribution (Normality Test). Then, the statistical test of the Student’s test was used to compare the two mean values for the independent samples. It is assumed that the null hypothesis is that the mean values of Young’s modulus are equal. Moreover, the alternative hypothesis is that the mean values of Young’s modulus are different. The null hypothesis assumes that any differences between the selected features that are noticeable are the result of chance. Therefore, an assumption is made that there is no difference between Young’s modulus; this is a demanded parameter in materials that proves that the material is isotropic. The statistical program Minitab was used for the calculations. The obtained *p*-value indicates at the unity level that (a) equals 0.05 and whether the null hypothesis can be assumed. It is estimated that if the value is higher, the probability that the normal distributions of the samples printed vertically and horizontally coincide is increased. Thus, it can be concluded that the material is isotropic.

**Figure 3:**
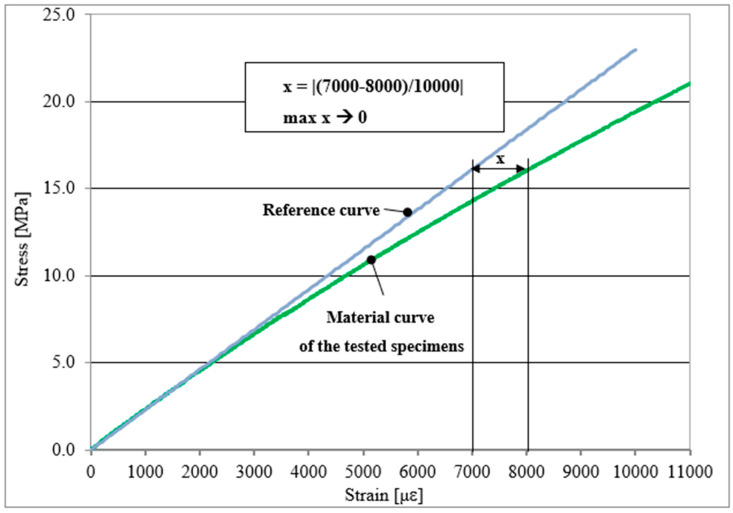
Scheme of linearity estimation; blue line—reference curve for specimens printed vertically or horizontally, green line—stress-strain material curve of the tested specimens.

The next important parameter is the estimation of the linearity of the material curve of the tested specimens due to the stabilization of the printing process and the choice of material for printing complex parts. An example of a scheme of linearity estimation is presented in Figure 3.

Hence, the relationships between the material stress–strain real and reference curves (engineering) are compared. For this purpose, a linear coefficient is determined. To properly designate the linear coefficient, each plot is transformed by the vector v [p, q], where p is the first reading of the strain gauge, and q is the calculated stress value. For each process, the linear coefficient is taken as the maximum value of tested samples, respectively, of raster orientations. In the range up to 10,000 μ, the maximum relative deviation between the deformations of the real curve (green line presented in Figure 3) and the reference line (blue line presented in Figure 3) is calculated, related to 10,000. The reference curve assumes a constant value of Young’s modulus. This Young’s modulus equals the arithmetic mean of three values which are determined during tests for specimens printed vertically and horizontally, respectively. The absolute value is determined, and the largest value is found, which is also the largest deviation from a straight line. The lower the value, the closer the material curve is to a straight line in a given range. The linear coefficient is adopted as the worst case in a given series, i.e., the maximum value of all tested specimens.

## 3. Results

A summary of geometric accuracy and mean values of cross-sectional thickness and width for five printing processes is presented in Table 1.

**Figure 4:**
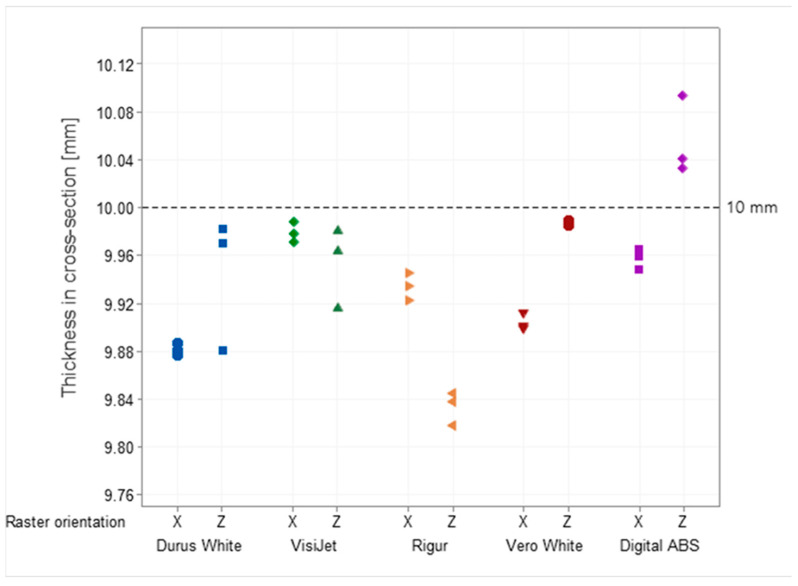
Results of cross-sectional thickness measurements obtained for Durus, VisiJet, Rigur, Vero, and ABS materials.

**Figure 5:**
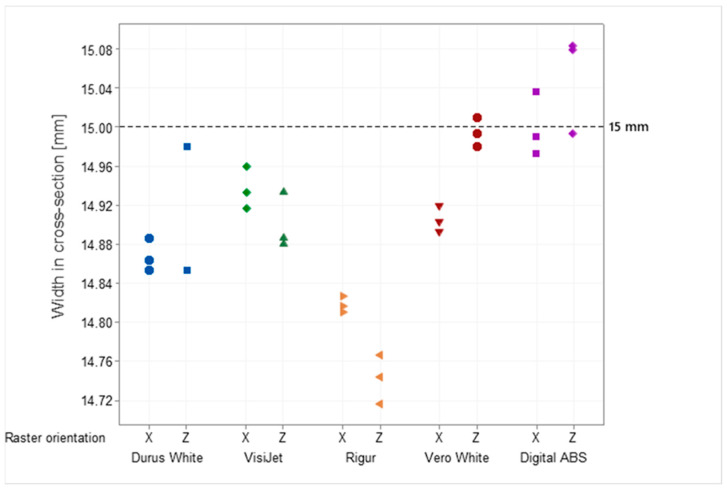
Results of cross-sectional width measurements obtained for Durus, VisiJet, Rigur, Vero, and ABS materials.

Specimens have a common feature which are areas with lower accuracy in the range from −0.447 mm (Durus White) to +0.411 mm (Digital ABS), depending on the material. The raster orientation does not have an influence on the deviation; therefore, horizontal printed specimens (X) are geometrically like the vertical ones (Z). The nominal dimensions in the CAD model are 10 mm for thickness and 15 mm for width. The mean value of the six specimens’ measurements is in the range of 9.88 mm (Rigur) to 10.01 mm (Digital ABS) for thickness and in the range of 14.78 mm (Rigur) to 15.03 mm (Digital ABS) for width depending on the material. The results of cross-sectional thickness and width measurements for each specimen are shown in Figure 4 and Figure 5, respectively.

Specimens printed from Durus White, VisiJet, Rigur, and Vero White materials in both vertical (Z) and horizontal (X) positions are characterized by a material deficit for width and thickness in the cross-section. Vero White specimens printed in the vertical position (Z) are characterized by very high accuracy close to the nominal size. Specimens printed in vertical (Z) and horizontal (X) positions with Rigur material have the lowest accuracy. Specimens printed in the vertical position (Z) from Digital ABS are characterized by an excess of material, and horizontal specimens (X) with a deficit.

A summary of mean values, standard deviation (SD), and coefficient of variation (CV) of tensile strength, Young’s modulus, Poisson’s ratio, linear coefficient, as well as *p*-value is gathered in Table 2.

Specimens printed from PJ technology characterize tensile strength in the range from 21 MPa (Durus White) to 43 MPa (Digital ABS). The lowest CV equals 5.6% observed for Digital ABS, and the highest CV equals 23.2% for Vero White. Poisson’s ratio for VisiJet, Digital ABS, Durus White, and Rigur materials is up to 2.8%. The value of Young’s modulus varies from 1.43 GPa (Durus White) to 2.85 GPa (Vero White), depending on the material. A wide range of *p*-values is observed, from 0.04 (VisiJet) to 0.59 (Rigur). Meanwhile, the mean linear coefficient has a narrow range, from 0.16 (Rigur) to 0.36 (Durus White), depending on the material.

**Figure 6:**
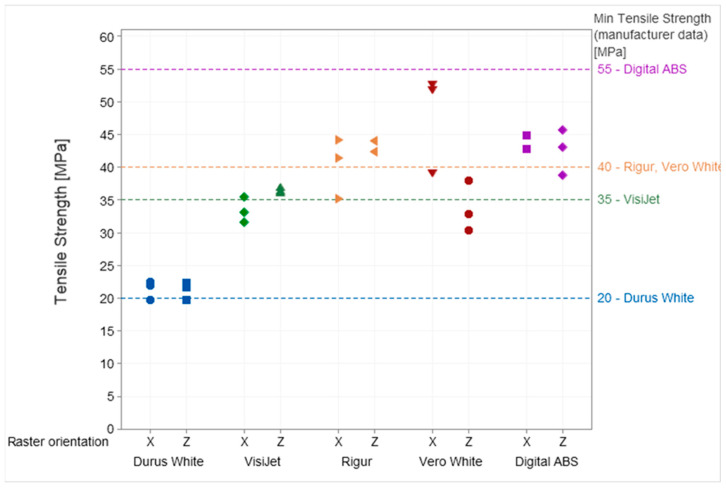
Results of tensile strength obtained during the test for Durus, VisiJet, Rigur, Vero, and ABS materials.

**Figure 7:**
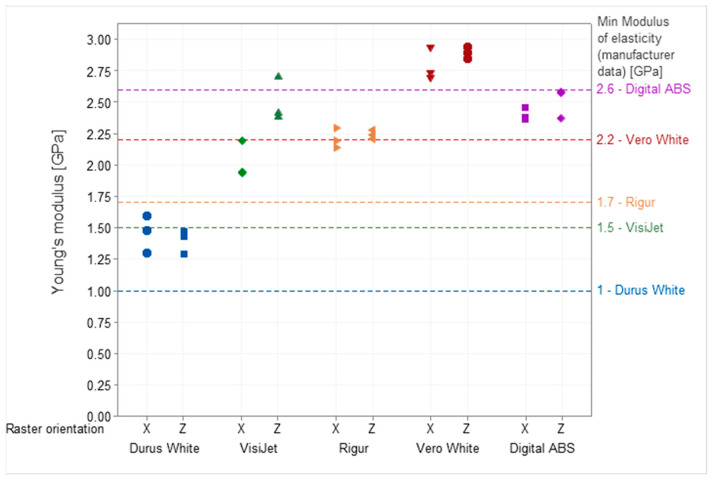
Young’s modulus was obtained during the test for Durus, VisiJet, Rigur, Vero, and ABS materials.

**Figure 8:**
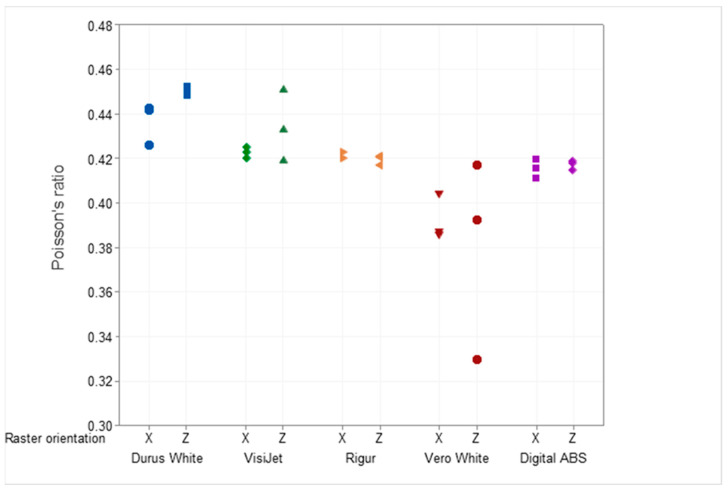
Poisson’s ratio was obtained during the test for Durus, VisiJet, Rigur, Vero, and ABS materials.

Additionally, the results for tensile strength (Figure 6) and mechanical properties of both Young’s modulus (Figure 7) and Poisson’s ratio (Figure 8) for each specimen are presented.

The raster orientation does not have an influence on the tensile strength of the specimens printed from Durus White, Rigur, and Digital ABS materials. The specimens printed horizontally (X) from VisiJet are characterized by lower tensile strength than vertical ones. Meanwhile, the specimens printed horizontally (X) from Vero White are characterized by higher tensile strength than vertical ones.

The raster orientation does not have an influence on Young’s modulus for the specimens printed from Durus White, Rigur, Vero White, and Digital ABS materials. The specimens printed horizontally (X) from VisiJet are characterized by lower Young’s modulus than vertical (Z) ones.

The raster orientation does not have an influence on Poisson’s ratio for the specimens printed from Rigur and Digital ABS materials. The specimens printed horizontally (X) from Durus White and VisiJet are characterized by a lower Poisson’s ratio than vertical (Z) ones. Specimens printed vertically (Z) from Vero White material are distinguished by the largest dispersion of Poisson’s ratio among all materials.

**Figure 9:**
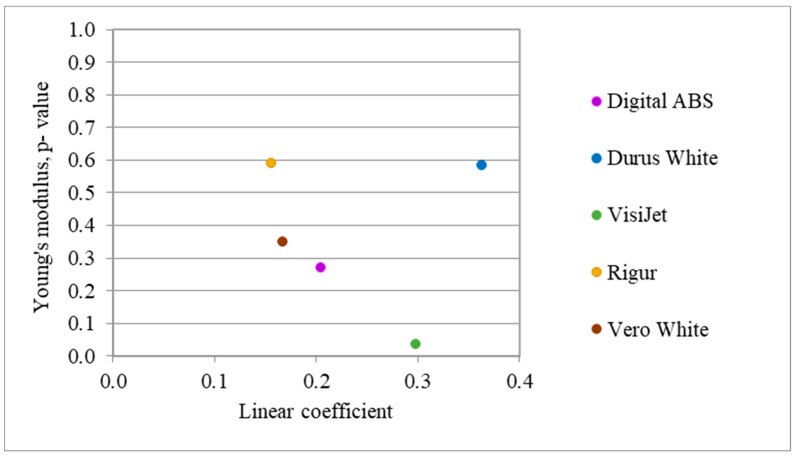
List of materials in terms of isotropy and linearity.

Figure 9 presents a list of the tested materials in terms of relative isotropy (*p*-value) and linear coefficient.

The expected material is a material that has both a high *p*-value and a low linear coefficient. Based on this, Rigur best meets this criterion. Vero White and Durus White materials meet only one condition for linear coefficient and *p*-value, respectively. On the one hand, Vero White material is distinguished by both a low linear coefficient and low *p*-value. On the other hand, Durus White is characterized by both a high *p*-value and a high linear coefficient. The Digital ABS has properties close to the Vero White. VisiJet is characterized by the lowest *p*-value and high linear coefficient.

**Figure 10:**
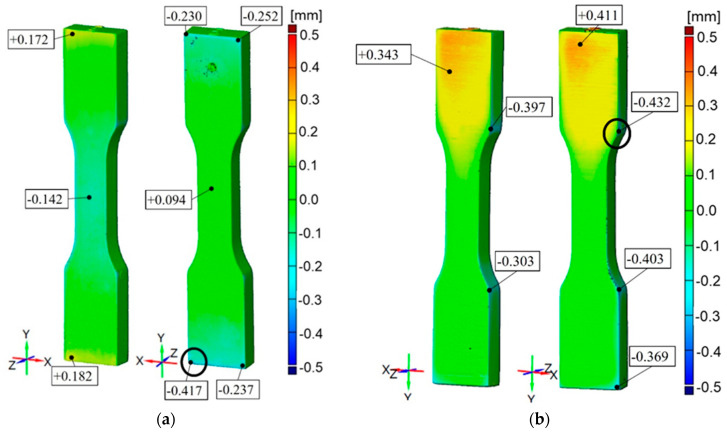
Geometric accuracy [mm] for the specimens made of ABS Ivory GEN 2 material and printed with nominal layer thickness 0.03 mm: (**a**) Raster orientation: horizontal (X-axis), specimen ID: 2; (**b**): Raster orientation: vertical (Z-axis), specimen ID: 5.

**Figure 11:**
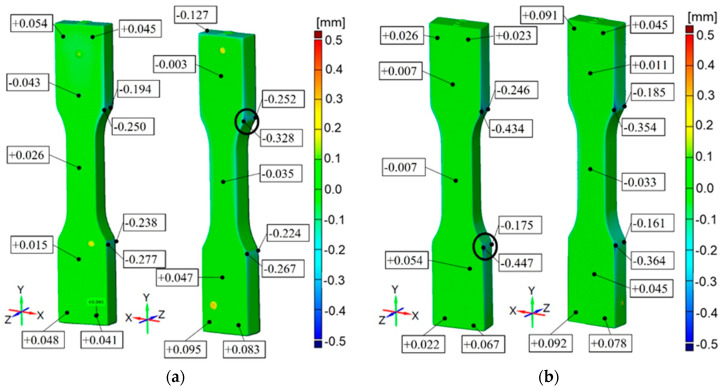
Geometric accuracy [mm] for the specimens made of Durus White material printed with nominal layer thickness 0.016 mm: (**a**) Raster orientation: horizontal (X-axis), specimen ID: 2; (**b**): Raster orientation: vertical (Z-axis), specimen ID: 5.

**Figure 12:**
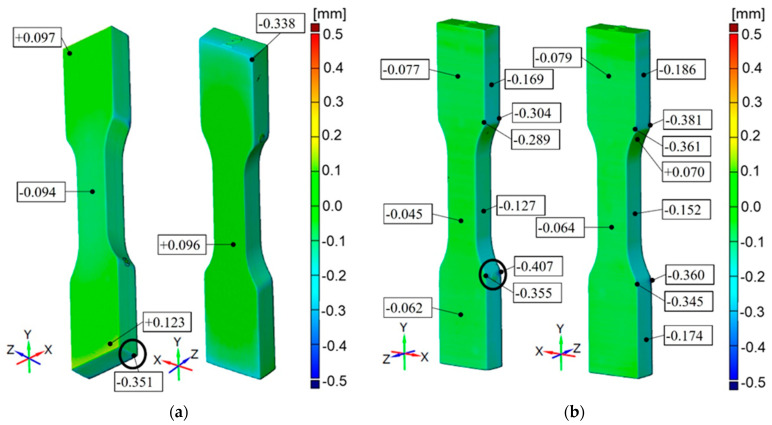
Geometric accuracy [mm] for the specimens made of Rigur material printed with nominal layer thickness 0.016 mm, Objet Connex3 3D printer: (**a**) Raster orientation: horizontal (X-axis), specimen ID: 2; (**b**): Raster orientation: vertical (Z-axis), specimen ID: 5.

**Figure 13:**
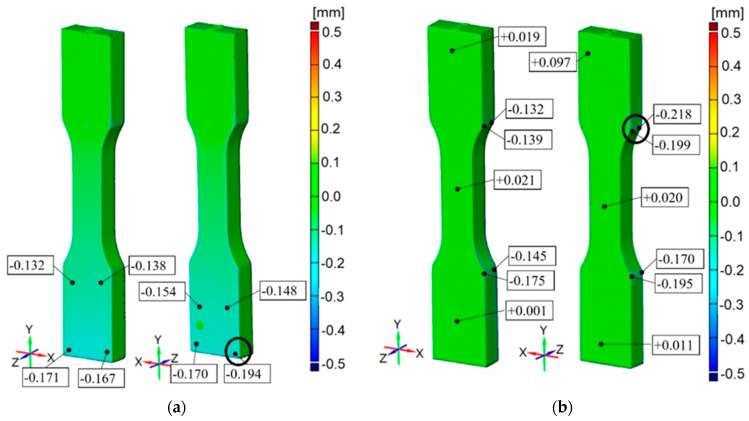
Geometric accuracy [mm] for the specimens made of Vero White Plus material: (**a**) Raster orientation: horizontal (X-axis), specimen ID: 2; (**b**): Raster orientation: vertical (Z-axis), specimen ID: 5.

**Figure 14:**
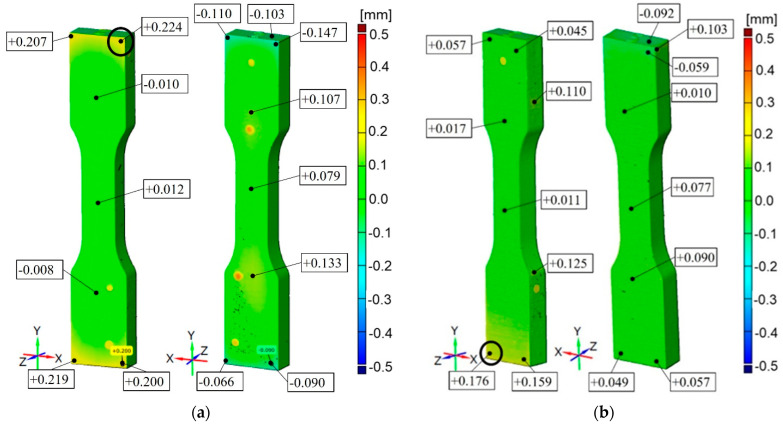
Geometric accuracy [mm] for the specimens made of VisiJet M2R-WT material: (**a**) Raster orientation: horizontal (X-axis), specimen ID: 2; (**b**): Raster orientation: vertical (Z-axis), specimen ID: 5.

Due to the shape of the specimens, views for each side are shown. In addition, specimen scans presented in this Section show plots from −500 mm to 500 mm to better compare results between processes (Figure 10, Figure 11, Figure 12, Figure 13 and Figure 14). Critical locations with the highest absolute value of deviation for the specimens are additionally marked with the black circle.

**Figure 15:**
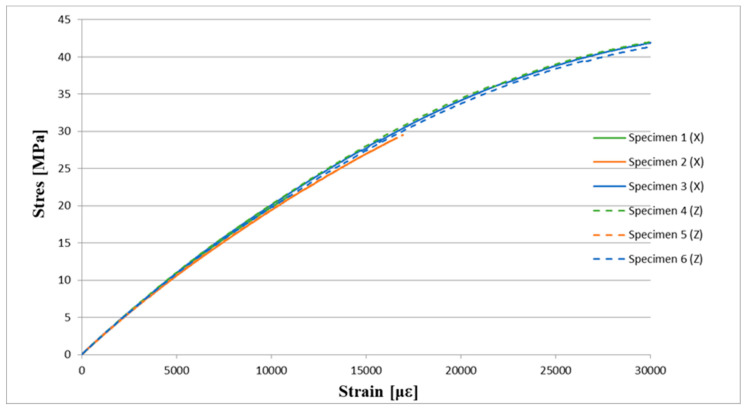
Stress-strain curves for six specimens made of Rigur material, three printed horizontally (X) and three vertically (Z).

**Figure 16:**
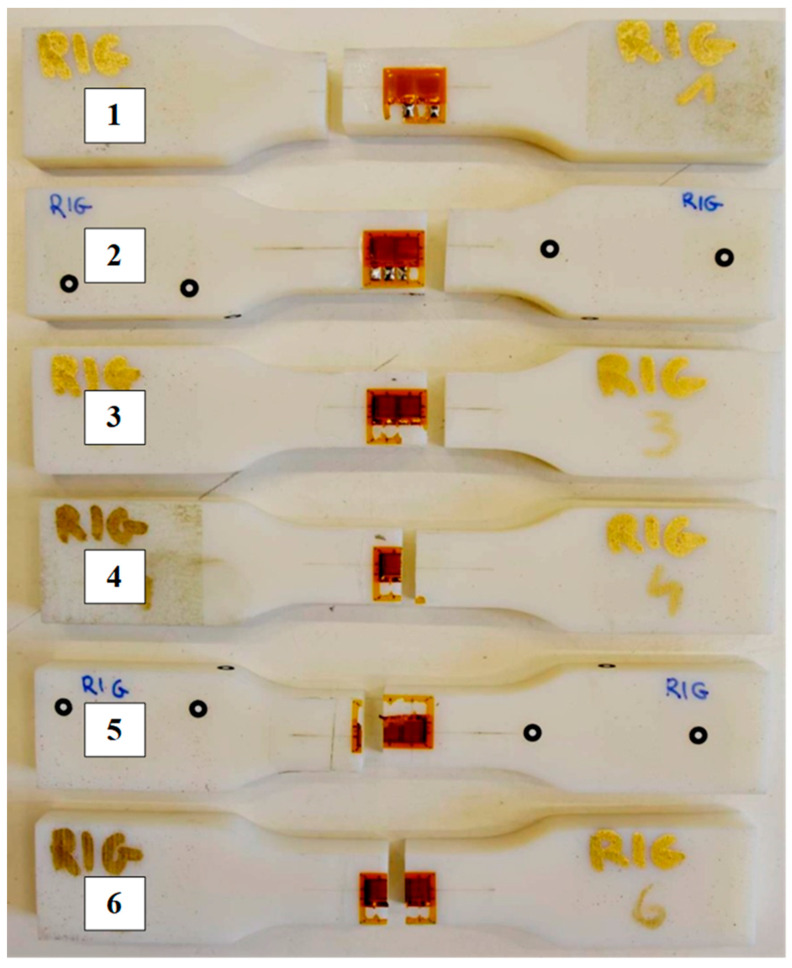
Six specimens made of Rigur material after tensile test, three (1–3) printed horizontally (X) and three (4–6) printed vertically (Z).

Detailed results are presented in Section 3.1 to Section 3.5. In addition, the results for Rigur are selected to be representative of all five processes due to their significance; therefore, details of this process (Figure 15 and Figure 16) are shown in Section 3.3.

### 3.1. Analysis for ABS Ivory GEN 2 Material

For ABS Ivory GEN 2, accuracy results are from −0.417 mm up to +0.172 mm for the part printed horizontally and from −0.432 mm to +0.411 mm for the part printed vertically. It can be observed that this is an excess of materials in the middle of the part up to +0.4 mm and a deficit of up to −0.4 mm at the edges (Figure 10).

In the cross-section of the specimens, the dimensions have deviations from the nominal values in the range of +0.1 mm to −0.05 mm. For the same deformations, the vertical specimens are characterized by higher stress values than the horizontal specimens. The tensile strength of the parts is significantly below the manufacturer data (55–60 MPa). The distribution of the modulus of elasticity shows that the tested parts do not reach the values specified by the manufacturer (2.6 GPa). Poisson’s ratio averaged 0.42 regardless of the specimen’s raster orientation.

### 3.2. Analysis of Durus White Material

For Durus White, accuracy results are from −0.328 mm up to +0.095 mm for the part printed horizontally and from −0.447 mm to +0.092 mm for the part printed vertically. The common feature for these specimens is the overall accuracy of printed elements equal to ±0.1 mm and some small areas with lower accuracy up to −0.4 mm (Figure 11).

In the cross-section of the specimens, the dimensions have a material deficit of −0.15 mm. The stress-strain curves overlap. This is not enabled to draw a conclusion about the trend resulting from the printing raster orientation. The distribution of the modulus of elasticity shows that the tested parts reached higher values than the manufacturer values (1–1.2 GPa). Therefore, in the study, the linearity of the curve in relation to the reference curve of 0.36 is the highest for all tested materials. Compared to the data provided by the manufacturer, there is higher deformation for the same stresses. The average tensile strength of the specimens, 21 MPa is in the lower range given by the manufacturer (20–30 MPa). Moreover, the tensile strength for specimens printed vertically (mean 21.4 MPa) and horizontally (mean 21.3 MPa) has a similar value. The obtained results are convergent with the statistical analysis in which the *p*-value equals 0.58. This means that it is highly probable that the Durus material has properties attributed to isotropic materials. Poisson’s ratio averaged 0.45 for vertically printed specimens and 0.44 for horizontally printed ones.

### 3.3. Analysis of Rigur Material

For Rigur printed on Objet Connex3 3D printer, accuracy results are from −0.351 mm up to +0.123 mm for the part printed horizontally and from −0.407 mm to +0.07 mm for the part printed vertically. The common feature for these specimens is the overall accuracy of printed elements equal to ±0.1 mm and some edge areas with lower accuracy up to −0.4 mm. In the cross-section of the specimens, the dimensions have a material deficit of −0.3 mm (Figure 12).

The stress–strain curves overlap (Figure 15). This is not enabled to draw a conclusion about the trend resulting from the printing raster orientation.

The strength is within the range specified by the manufacturer (40–45 MPa) for the vertical specimens and is within this range or below the lower limit of the range for horizontal specimens. The distribution of the modulus of elasticity shows that the tested parts are above the range specified by the manufacturer (1.7 GPa). Poisson’s ratio averaged 0.42 regardless of the specimen’s raster orientation. The broken specimen after the test is presented in Figure 14.

Observations of specimens (Figure 16) reveal that the crack goes as expected in the narrow cross-section of specimens. Moreover, for specimens printed vertically (4–6), the crack goes in almost the same place in the center of specimens. For each vertically printed specimen, the crack is at the place of the strain gauge. However, in the case of horizontally printed specimens (1–3), the crack is located below or above the strain gauge. Additionally, macrostructure observations indicate straight and smooth crack edges for each specimen. The raster orientation does not have an influence on the fracture shape.

### 3.4. Analysis of Vero White Plus Material

For Vero White Plus, accuracy results are from −0.194 mm up to +0.025 mm for the part printed horizontally and from −0.218 mm on the edges to +0.097 mm for the part printed vertically. The common feature for these specimens is some areas with a material deficit of up to −0.2 mm and material surplus of up to +0.1 mm (Figure 13).

In the cross-section of the specimens, the dimensions have a material deficit of −0.1 mm. For the same deformations, despite the material, curves overlap. It can be observed that the vertical specimens are characterized by higher stress values than the horizontal specimens. The tensile strength is below the range specified by the manufacturer (40–55 MPa) for the vertical specimens and slightly below the lower limit for the horizontal specimens. The distribution of the modulus of elasticity shows that the tested parts are in the range specified by the manufacturer (2.2–3 GPa). The Vero White material has the greatest spread and is the most unpredictable material of all the tested.

### 3.5. Analysis for VisiJet M2R-WT Material

For VisiJet M2R-WT (PJ method), accuracy results are from −0.147 mm to +0.224 mm for the part printed horizontally and from −0.092 mm to +0.176 mm for the part printed vertically. The common feature for these specimens is the overall accuracy of printed elements equal to ±0.1 mm and some small areas with lower accuracy up to ±0.2 mm (Figure 14).

In the cross-section of the specimens, the dimensions have a material deficit of −0.12 mm. For the same deformations, the vertical specimens are characterized by higher stress values than the horizontal specimens. The tensile strength of vertical parts is within the range specified by the manufacturer (35–45 MPa), and horizontal parts are slightly below the lower limit. The distribution of the modulus of elasticity shows that the tested parts are above the range specified by the manufacturer. Poisson’s ratio averaged 0.43 for vertically printed specimens and 0.42 for horizontally printed ones.

## 4. Discussion

The comparison of both materials and methods for the Material Jetting technology based on the conducted test has been presented in this paper.

Geometric analysis of the reference specimens demonstrated slight differences between the MJ and PJ methods. The accuracy of the print within the selected materials, technology, and printing direction turned out to be very similar and amounts to ±0.2 mm. Furthermore, the layer thickness does not significantly improve the accuracy of the models.

It is concluded that printed models have a common feature, which is the unitary surface deviation with general dimensional accuracy equal to ±0.1 mm with some small areas with a lower accuracy depending on the material and printer device (Figure 10, Figure 11, Figure 12, Figure 13 and Figure 14). Comparing the specimens, the conclusion can be drawn that one of the most important parameters determining the correctness of the printouts is the ability to properly remove the support material. Material losses observed in all specimens at the edges may result from mechanical interference in the geometry of the specimens during the removal of the support material. It can be observed that the horizontal edges of the specimen are more deviating and susceptible to geometric deviations than the vertical edges of the specimen. The general view of the results draws the conclusion that horizontal printed specimens are geometrically like vertical ones.

The PJ method is characterized by the high accuracy and resolution of printed models. For those specimens, some small areas on the edges with lower accuracy are up to −0.45 mm. The smallest geometric inaccuracy for the PJ method is observed for Vero White Plus material, where the deficit of materials is up to −0.2 mm (Figure 13). Additionally, Vero White Plus has the highest spread in terms of tensile strength and is the most unpredictable of all the materials tested. However, the biggest geometric inaccuracy for ABS material is noticed, where both deficit of materials up to −0.4 mm and excess of materials in the middle of the part up to +0.4 mm are (Figure 10). On the other hand, taking the dimensions cross-section of the specimens into account, Digital ABS has the closest values compared with the nominal ones (Figure 4 and Figure 5).

The nominal accuracy has been confirmed for each case. Therefore, the most important criterion in choosing a material is the result of tensile strength tests. The main comparative criterion is the isotropy of the material, where the distributions of the modulus of elasticity and the possibly linear characteristics of the material curves are compared. According to Figure 9, Rigur, which is the closest to the point (0,1), has the expected properties. On the other hand, VisiJet material does not meet the criteria set out in this research due to the lowest p-value and high value of the linear coefficient.

One of the most important parameters to be compared in this article is to check the correlation between specimens printed in two directions. The greater the *p*-value, the greater the probability that the normal distributions of specimens printed vertically and horizontally coincide to a greater extent. Therefore, it can be considered that the material behaves isotopically. Due to the *p*-value parameter, for models printed with Rigur and Durus materials, the direction of printing is irrelevant. However, the Durus material is also characterized by the highest value of the linear coefficient, which means that it will most likely be an issue to obtain a stable and repeatable printing process.

To summarize, the best results from all five processes are from Rigur material. There is a high probability that this material will be suitable for printing and testing complex geometries, where the isotropy of the material is an important parameter, including the entire complex 3D model.

No repetition of tests for an increased number of specimens or complex shapes is limited for this study. Nowadays, the matter of accuracy, the printing resolution comparison, and the research of material properties are extensively studied. The determined mechanical values can be used for further analysis of Rigur materials and for the production of complex 3D models. Most of the investigations have not been released yet.

## Figures and Tables

**Table 1:** Summary of geometry accuracy deviation of specimens for PJ and MJ methods.

Material	Raster Orientation ^(1)^	Deviation [mm]		Mean Value [mm]	
		Min	Max	Thickness	Width
VisiJet	X	−0.147	+0.224	9.97	14.92
	Z	−0.092	+0.176		
Digital ABS	X	−0.417	+0.172	10.01	15.03
	Z	−0.432	+0.411		
Vero White	X	−0.194	+0.025	9.95	14.95
	Z	−0.218	+0.097		
Durus White	X	−0.328	+0.095	9.91	14.9
	Z	−0.447	+0.092		
Rigur	X	−0.351	+0.123	9.88	14.78
	Z	−0.407	+0.07		

Note: ^(1)^ specimens printed in two directions: vertically—along the Z axis (Z), and horizontally—along the X axis (X).

**Table 2:** Summary of mechanical properties of specimens for PJ and MJ methods.

Material	Tensile Strength			Poisson’s Ratio			Young’s Modulus			*p*-Value [0–1]	Mean Linear Coefficient [0–1]
	Mean Value [MPa]	SD ^(1)^	CV ^(2)^ [%]	Mean Value [-]	SD ^(1)^	CV ^(2)^ [%]	Mean Value [GPa]	SD ^(1)^	CV ^(2)^ [%]		
VisiJet	35	2.1	6	0.43	0.012	2.8	2.26	0.298	13.2	0.04	0.3
Digital ABS	43	2.4	5.6	0.42	0.003	0.8	2.46	0.1	4.1	0.27	0.2
Vero White	41	9.49	23.2	0.39	0.03	7.8	2.85	0.1	3.6	0.35	0.17
Durus White	21	1.25	5.9	0.44	0.01	2.2	1.43	0.12	8.2	0.58	0.36
Rigur	42	3.49	8.3	0.42	0.002	0.5	2.23	0.06	2.6	0.59	0.16

Notes: ^(1)^ standard deviation (SD); ^(2)^ coefficient of variation (CV).

## Data Availability

The data that support the findings of this study are available from the corresponding author upon reasonable request.
